# INC280 inhibits Wnt/β-catenin and EMT signaling pathways and its induce apoptosis in diffuse gastric cancer positive for c-MET amplification

**DOI:** 10.1186/s13104-019-4163-x

**Published:** 2019-03-11

**Authors:** Sung-Hwa Sohn, Bohyun Kim, Hee Jung Sul, Yoo Jin Kim, Hyeong Su Kim, Hongtae Kim, Jong Bok Seo, Youngho Koh, Dae Young Zang

**Affiliations:** 1 Hallym Translational Research Institute, Hallym University Sacred Heart Hospital, Anyang, 14066 Republic of Korea; 2 Division of Hematology-Oncology, Department of Internal Medicine, Hallym University Medical Center, Hallym University College of Medicine, 22, Gwanpyeong-ro 170beon-gil, Dongan-gu, Anyang-si, Gyeonggi-do 14086 Republic of Korea; 3 Korea Basic Research Institute Seoul Center, Seoul, 02855 Republic of Korea; 4 Department of Bio-medical Gerontology, Ilsong Institute of Life Sciences, Hallym University, Anyang, Gyeonggi-do 14066 Republic of Korea; 5 School of Life Sciences, Ulsan National Institute of Science and Technology, Ulsan, 689-798 Republic of Korea

**Keywords:** INC280, Gastric cancer, c-MET, RUNX3, Diffuse type

## Abstract

**Objective:**

Gastric cancer is more open related to genetic predisposition. In our RNA sequencing study on gastric cancer patients, Runt-related transcription factor-3 (RUNX3) expression was significantly down-regulated in gastric cancer. We showed that decreased levels of RUNX3 are significantly associated with c-MET (r = − 0.4216, *P *= 0.0130). In addition, c-MET expression is a candidate for targeted therapy in gastric cancer. Therefore, in the present study, the anti-cancer effects of the c-MET inhibitor on gastric cancer cells from positive or negative for c-MET amplification were evaluated.

**Results:**

INC280 treatment inhibits growth of a c-MET-amplified MKN45 (RUNX3-positive) and SNU620 (RUNX3-negative) diffuse type cells. Then, INC280 showed the highest inhibition and apoptotic rates with the lowest IC_50_s in MKN45 cells but not in c-MET-reduced MKN28 (intestinal type) cells. We also showed that INC280 inhibits the WNT signaling pathway and SNAIL expression in MKN45 cells. The data indicate that INC280 could be used as therapeutic agents for the prevention or treatment of diffuse gastric cancer positive for c-MET amplification.

**Electronic supplementary material:**

The online version of this article (10.1186/s13104-019-4163-x) contains supplementary material, which is available to authorized users.

## Introduction

High-incidence areas of GC include Eastern Europe, South America, and East Asia [1–3]; however, the mortality rates have decreased markedly in recent years [4]. In Korea, despite a decline in the incidence of GC, it remains the second most common cancer [5]. In addition, unsatisfactory treatment outcomes are caused by differences in the histological classifications of GC between the intestinal- and diffuse-type. Intestinal-type GC is related to *Helicobacter pylori* infection and diffuse-type GC is more open related to genetic predisposition. Thus, diffuse-type is less related to environmental factors [6]. To overcome this problem, and to develop and identify new drug candidates, determining tumor characteristics and treatment parameters is important.

The Wnt/β-catenin signaling pathway and EMT are associated with a wide range of GC progression events. EMT was observed in the invasive progression of cancer that initiates diffuse GC in the absence of hyperproliferation and β-catenin activation [7]. SNAIL, a key transcriptional repressor of E-cadherin expression, is a well-known trigger of EMT, leading to irreversible tumorigenesis in mice [8]. Recent studies have revealed that downregulation of the proto-oncogene MET suppresses EMT in prostate cancer [9]. Moreover, MET amplification is a frequent molecular abnormality in GC [10, 11].

In the present study, we applied an RNA-seq approach to identify MET and RUNX3 genes differentially expressed in the GC and adjacent normal tissues from 34 patients. We evaluated the effects of INC280 on the suppression of GC proliferation, migration, and apoptosis according to Lauren’s classification.

## Main text

### Methods

#### Materials

INC280 was supplied from Novartis (Basel, Switzerland). The compounds were dissolved in dimethyl sulfoxide at 10 mmol/L prior to use in all in vitro studies.

#### Human gastric tissue specimen collection

The GC and adjacent normal tissues obtained from 34 patients were approved by the Ethics Committee of Hallym University Sacred Heart Hospital (2015-I078) and were selected as the discovery cohort for RNA-seq. Additional file 1: Table S1 summarizes the discovery sets.

#### RNA-seq analysis

RNA-seq experimental procedures were performed using standard procedures. The raw reads were saved in the FASTQ format, and the dirty raw reads were removed before analyzing the data. Reads that could be uniquely mapped to a gene were used to calculate the gene expression levels, which were measured based on the number of reads per kilobase of transcript per million mapped reads. We identified differentially expressed genes between paired tumor and normal samples, and a *P* value ≤ 0.001 was deemed to indicate statistical significance.

#### Cell lines and cell culture

The human GC cell lines SNU5, SNU16, SNU620, MKN7, MKN28, MKN45, MKN74, AGS, and KATO-III were obtained from the KCLB (Seoul, Korea). Cell culture was performed using standard procedures.

#### Growth inhibition assays

The IC_50_ values of INC280 on SNU620, MKN28 and MKN45 cells were measured using the MTS assay for the selected drugs at concentrations of 10, 1, 0.1, 0.05, 0.0025, 0.00125, 0.001, 0.0001, 0.00001 or 0.000001 µM for 48 h. On the day of the proliferation assay, the medium was removed, and 200 µL of fresh medium was added to each well of the 96-well plates, followed by 20 µL of MTS solution, and the plates were incubated at 37 °C for 2 h in a humidified environment with 5% CO_2_. The absorbance was read at 490 nm using a Synergy-2 Multi-Mode Microplate Reader (BioTek). The IC_50_ values were determined after fitting growth inhibition curves to dose–response curves using GraphPad Prism software (GraphPad Software Inc., USA).

#### Cell migration assay

MKN28 and MKN45 cells were diluted and seeded at a density of about 1 × 10^5^ cells per well in 6-cm plates. After incubation for 1 day, a straight scratch was made on the cells using a P200 pipette tip. The cells were then washed with phosphate-buffered saline and were further cultured with or without INC280 in RPMI1640. After incubation for 0, 24, and 72 h, the gap width of the scratch re-population was photographed and then compared with the initial gap size at 0 h.

#### Apoptosis analysis

The MKN28, SNU620, and MKN45 cells seeded into 6-well plates at a density 5 × 10^4^ cells per millilitre were treated with IC_50_ values of INC280. Cell death was determined using the annexin V-APC/PI apoptosis detection kit (Thermo Fisher Scientific, USA) using a CytoFLEX flow cytometer (Beckman Coulter, USA). The percentage of intact and apoptotic cells were calculated using CytExpert software (Beckman Coulter).

#### qRT-PCR analysis

To quantitate mRNA expression, the total RNA from each sample was reverse-transcribed into cDNA using the High Capacity cDNA reverse Transcription Kit (Applied Biosystems, USA). qRT-PCR was performed using the Power SYBR Green PCR Master mix and a LightCycler 96 instrument (Roche Applied Science, USA). The transcript levels of GAPDH were used for sample normalization. Primer sequences are listed in Additional file 1: Table S2.

#### Immunoblot analysis

Immunoblot analysis was performed using standard procedures. The antibodies are listed in detail in Additional file 1: Table S3.

#### Immunofluorescence microscopy

MKN45 cell cultured on chamber slides were washed with PBS and fixed with 4% paraformaldehyde, after which they were incubated with anti-β-catenin monoclonal antibody (BD Transduction Laboratories) and stained with anti-mouse IgG Alexa Fluor 488 (Invitrogen). Cells were examined using a ZEISS LSM700 confocal laser scanning microscope (Carl Zeiss, Oberkochen, Germany).

#### Statistical analysis

The data were statistically analyzed using Prism 5. All values are presented as mean ± standard error of the mean. Statistical significance was examined using the Mann–Whitney test and Fisher’s exact test. The Kaplan–Meier method was used to estimate OS, and differences between genotypes were compared by using the log-rank method. A *P* value < 0.05 was deemed to indicate statistical significance.

### Results

#### Baseline characteristics

**Fig. 1:**
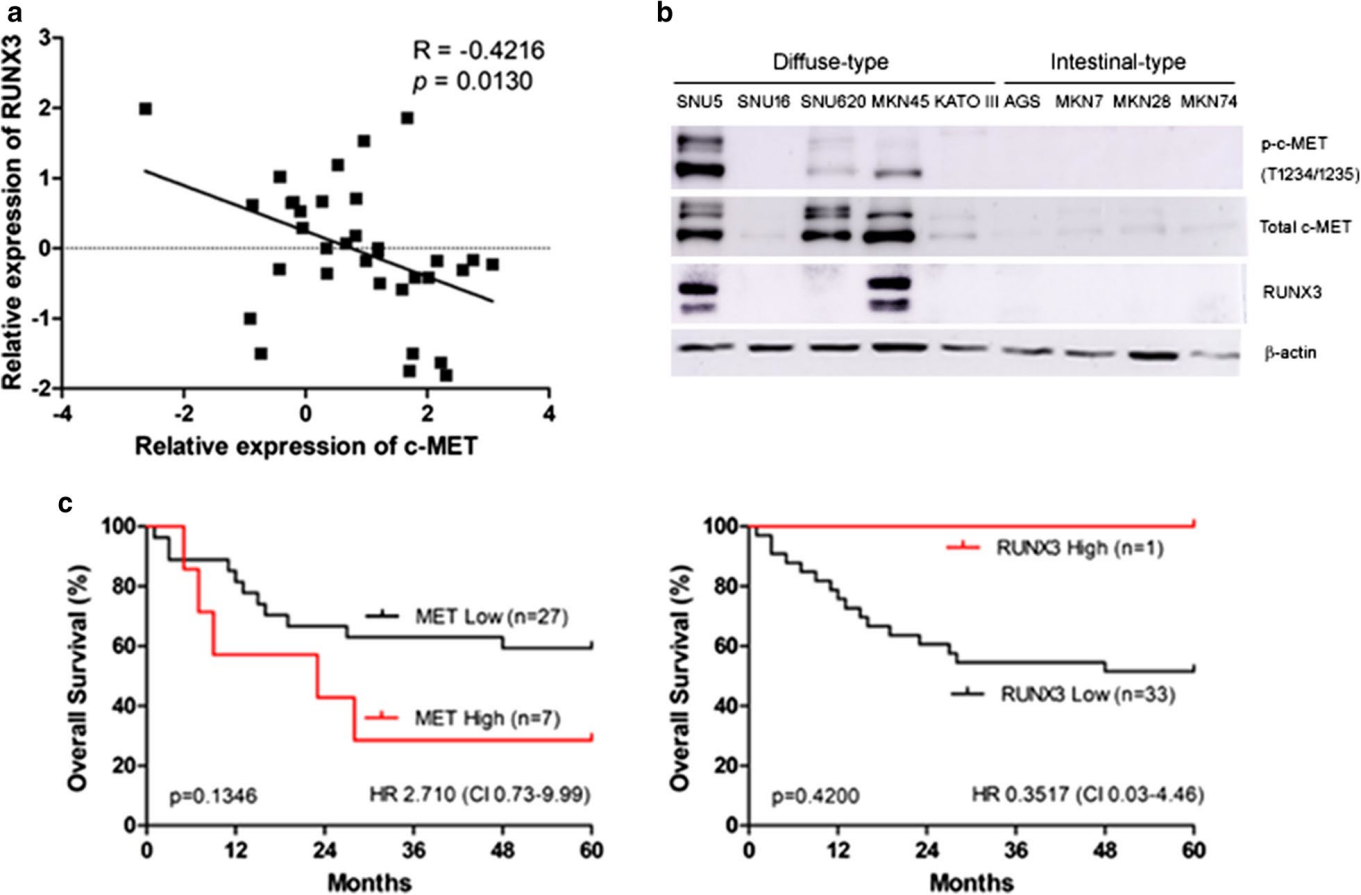
Correlation between RUNX3 levels and c-MET levels in gastric cancer patients. **a** Clinical significance of decreased RUNX3 expression and increased MET expression in gastric tumors. Expression of RUNX3 and MET mRNA in gastric cancer and normal pair samples as determined by RNA-sequencing. R = − 0.4216, P = 0.0130 by Spearman correlation. **b** Immunodetection of endogenous c-MET, phosphor c-MET (pY1234/1235) and RUNX3 in diffuse type- and intestinal type-gastric cancer cell lines. **c** Overall survival (OS) of patients with MET- or RUNX3-amplified tumors compared with low amplification

Thirty-four subjects were enrolled in the genetic alterations study using RNA-seq, and we identified differentially expressed genes such as MET and RUNX3. The associations of MET and RUNX3 expression with clinicopathological characteristics are shown in Additional file 1: Table S1. We found a correlation between the down-regulation of RUNX3 and MET overexpression (Fig. 1a), whereas only one (SNU620) cells showed a correlation pattern (Fig. 1b). Low expression of RUNX3 was significantly associated with poor differentiation (52.9%; *P *< 0.001), high expression of Ki-67 (79.4%; *P *< 0.001), diffuse-type histology (41.2%; *P *< 0.001) and recurrence (100%; *P *< 0.001) (Additional file 1: Table S1). OS analysis comparing contribution of MET or RUNX3 genotype of GC showed no statistically significant differences (log-rank *P* = 0.1346 and *P* = 0.4200, respectively; Fig. 1c). In this study, MET amplification doesn’t associated with a poor outcome. It is probably because MET amplification is present in 2–20% of GC patients, however, only 7% of tumors overexpressed p-MET in overexpressed MET [12]. p-MET was significantly associated with poor outcome [13].

#### Determining the effective dose of INC280 in intestinal and diffuse-type cells

**Fig. 2:**
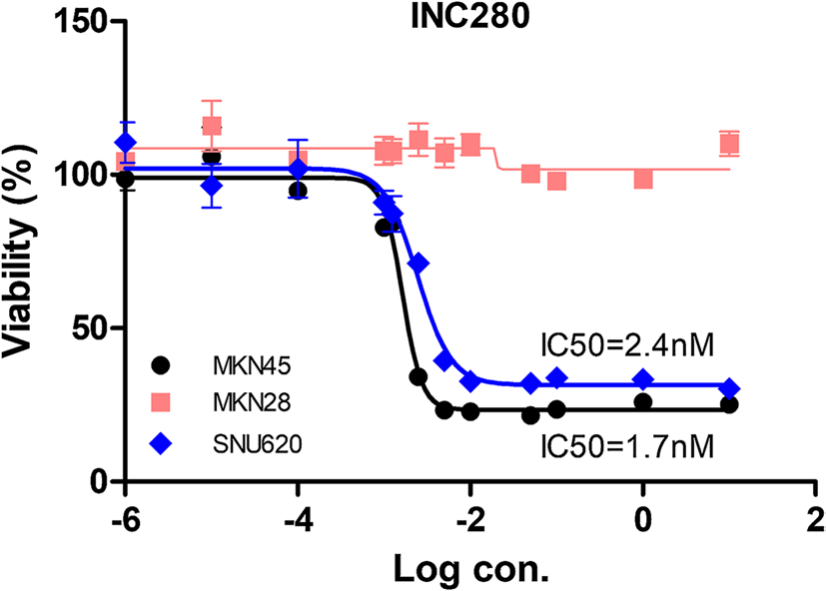
Effect of INC280 in c-MET amplified gastric cancer cells positive or negative for RUNX3 expression. MKN28, SNU620, and MKN45 cells were treated with various concentrations of INC280 for 48 h. The values of cell viability were then normalized against that of the control

To investigate the effect of the INC280 on c-MET amplified cell with or without RUNX3, c-MET and RUNX3 protein expression was evaluated in GC cells. c-MET and RUNX3 proteins were expressed in SNU5 and MKN45 cells. Interestingly, p-MET amplified gastric cancer cell lines belong to the diffuse-type (Fig. 1b). We tested the dose-dependent inhibitory effects of INC280 in MKN28 (intestinal-type), SNU620 (RUNX3-negative diffuse-type), and MKN45 (RUNX3-positive diffuse-type) cells (Fig. 2). The cells were treated with different concentrations of INC280 for 48 h, and the optimal dose was determined by evaluating the cell viability using MTS assays. Treatment with the INC280 decreased the cell viability in a dose-dependent manner in c-MET-amplified SNU620 and MKN45 cells but not in c-MET-reduced MKN28 cells (n = 3) (Fig. 2). The IC_50_ value of INC280 was determined using non-linear regression analysis [IC50 = 1.7 nM (MKN45) or 2.4 nM (SNU620)].

#### Effects of INC280 on cell migration

To determine the inhibitory effects of INC280 on MKN28 and MKN45 cells, cell migration was examined by wound-healing assay using the respective IC50 values of INC280 (Additional file 2: Figure S1). The wound gaps in the c-MET-amplified MKN45 cells treated with INC280 were significantly wider than those of treated c-MET-reduced MKN28 cells. INC280 showed an inhibitory effect on the c-MET-amplified cell line.

#### Effects of INC280 on cell apoptosis

To evaluate the effects of INC280 on cell death in MKN28, SNU620, and MKN45 cells, apoptosis was examined by staining with annexin V-APC/PI, followed by flow cytometry (Additional file 2: Figure S2). Cells were stained with annexin V-APC and PI, which assess early apoptotic and late apoptotic cell populations, respectively. INC280 showed the best cell death rates in SNU620 and MKN45 cells but not in MKN28 cells. The percentage of apoptotic cells was 22.59% and 23.56 ± 1.08% after exposure to INC280 for 48 h, respectively. By contrast, these drugs were inactive against MKN28 cells, which express low levels of c-MET.

#### INC280 inhibits c-MET activation and WNT/β-catenin signaling in RUNX3-positive diffuse-type cells

**Fig. 3:**
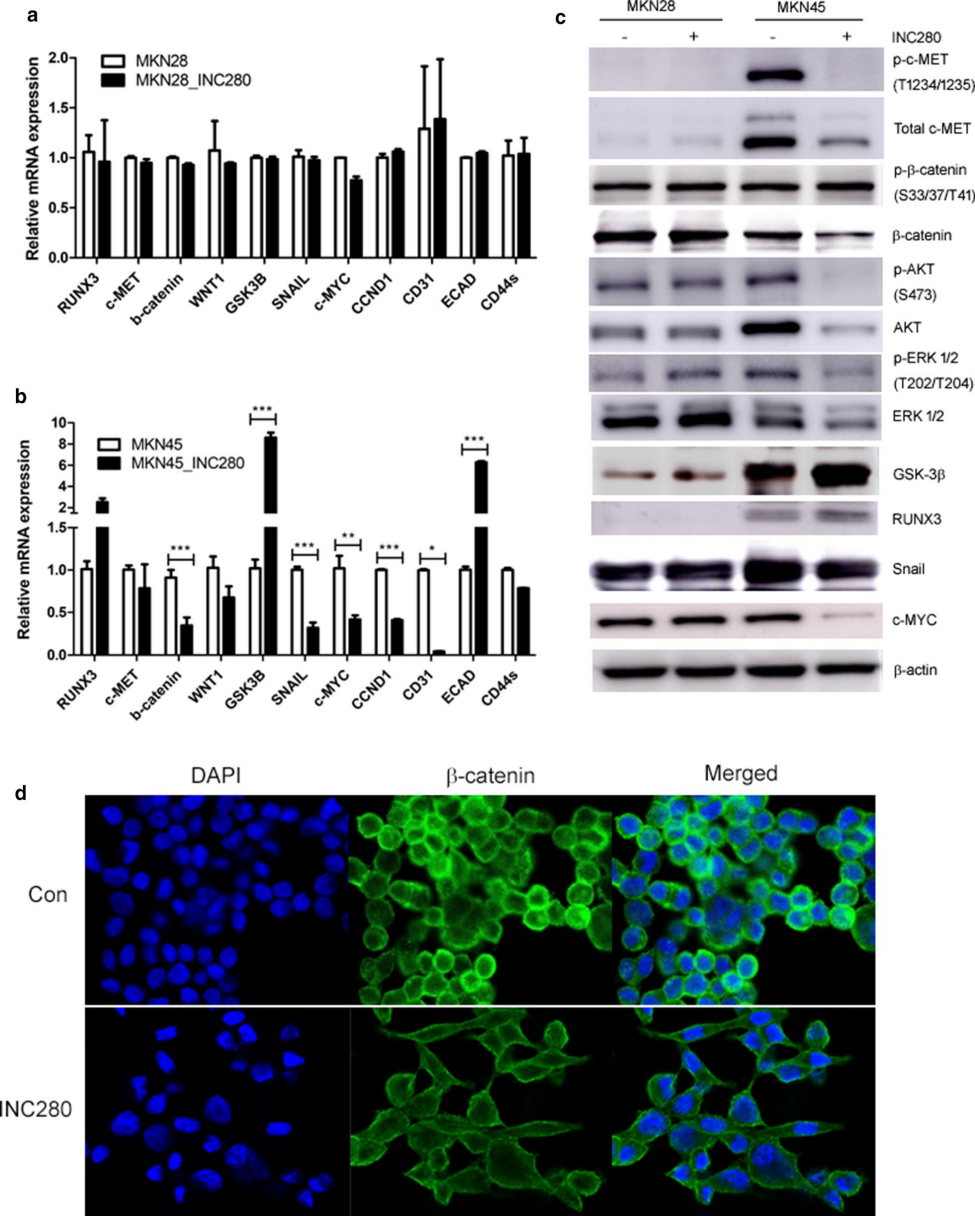
Effect of c-MET downregulation on RUNX3, SNAIL and the Wnt/β-catenin signaling pathway. mRNA expression of c-MET-RUNX3-regulated genes in MKN28 (**a**) and MKN45 (**b**) cells. **c** Protein levels of c-MET-RUNX3-regulated genes in MKN28 and MKN45 cells. ***p < 0.001. **d** A presentative confocal microscopy showing decreased of membrane bound β-catenin in INC280 treated MKN45 cells

To examine the inhibitory effects of INC280 on MKN28 and MKN45 cells, oncogenic pathways were examined by analyzing gene and protein expression (Fig. 3a–c). When cells were treated with INC280, the levels of GSK3β and E-cadherin were increased in MKN45 cells; by contrast, the levels of total c-MET, phosphorylated c-MET, β-catenin, Wnt/β-catenin downstream target gene (c-MYC, CCND1), angiogenic marker (CD31), and EMT marker (SNAIL) were decreased. However, these drugs were inactive against MKN28 cells. Confocal microscopy analysis of the cellular distribution of endogenous β-catenin on MKN45 cells showed a membranous staining pattern (Fig. 3d). Membranous β-catenin was highly reduced after 20 h of INC280 treatment. Surprisingly, treatment of MKN45 cells with INC280 that resulted in increased cell size. In mammalian cells, cell size increases when cell cycle progression is blocked [14]. INC280 may induce apoptosis and cell cycle arrest by degradation of cytosolic β-catenin.

### Discussion

Cancer is a disease caused by genetic alterations. Therefore, we conducted RNA-seq in GC samples and their matched adjacent normal tissues to identify genetic alterations. The RNA-seq study showed that decreased levels of RUNX3 were significantly associated with c-MET, however cell lines were no correlated between RUNX3 and c-MET. RUNX3 loss is an early event in GC progression due to aberrant Wnt/β-catenin signaling [15–17], which mediates EMT in GC [18], a process whereby epithelial cells are converted into migratory and invasive cells [19, 20].

c-MET amplification is present in 2–20% of GC and is associated with a poor outcome [10, 11, 21, 22]. c-MET is known to activate cancer cell proliferation, migration and tumor invasiveness [23, 24]. Recent preclinical studies have revealed that c-MET inhibitors, including onartuzumab, foretinib, crizotinib, and PHA-665752, have failed in clinical trials in GC patients [25, 26]. Therefore, identifying an appropriate c-MET-positive GC group that is sensitive to c-MET inhibition is an urgent issue. Therefore, in the present study, c-MET inhibitor drugs were screened to determine whether they could be used as therapeutic agents for the treatment of GC using growth inhibition assays of MKN45 cells. Among the six c-MET inhibitor drugs tested, INC280 showed high inhibitory activity; therefore, this drug was selected for further study. INC280 works against putative c-MET-dependent tumor types [27]. In the present study, INC280 showed increased inhibition and apoptotic rates, indicating therapeutic utility in MKN45 cells. When MKN45 cells were treated with INC280, the levels of total c-MET, phosphorylated c-MET, total β-catenin, CCND1, c-MYC, CD31, and SNAIL proteins or genes were decreased. By contrast, the levels of phosphorylated β-catenin, RUNX3, E-cadherin, and GSK-3β were increased. Our results additionally indicated that INC280 may suppress EMT through decreasing SNAIL expression. Indeed, our results showed that INC280, as well as GSK3β-mediated signaling, inhibits Wnt/β-catenin signaling by inhibiting c-MET phosphorylation in diffuse GC.

The results of this study indicate that c-MET and RUNX3 are differentially expressed in GCs compared with normal adjacent gastric mucosa and found a correlation between low RUNX3 levels and c-MET overexpression and tumor recurrence. INC280 shows significant inhibitory activity in c-MET-expressed diffuse GC. Our in vitro study strongly supports the clinical evaluation of INC280, which prevents c-MET-associated GC.

## Limitations

Our study reports an association between MET and diffuse-type. However, the lack of significance in our study could be due to relatively small sample size. Although diffuse GC positive for c-MET amplification might serve as a predictor for poor outcome, it is considered as actionable target.

## Additional files

**Additional file 1: Table S1.** Association of MET and RUNX3 expression with clinicopathological characteristics in 34 gastric cancer patients. **Table S2.** List of oligonucleotides for real-time PCR. **Table S3.** List of antibodies and its characteristic.

**Additional file 2: Figure S1.** Wound-healing assay was used to assess the effect of INC280 on the migration ability of MKN28 and MKN45 cells. INC280-treated MKN45 cells showed suppressed migration ability compared with INC280-treated MKN28 cell lines. **Figure S2.** Effect of INC280 on cell death in MKN28, SNU620, and MKN45 cells. Flow cytometric analysis of INC280-induced apoptosis in A: MKN28 and MKN45, B: SNU620 cells. PI, propidium iodide.

## Abbreviations

GC: gastric cancer
EMT: epithelial–mesenchymal transition
RUNX3: Runt-related transcription factor-3
RNA-seq: RNA sequencing
KCLB: Korean Cell Line Bank
PBS: phosphate buffered saline
PI: propidium iodide
MTS: 3-(4,5-dimethylthiazol-2-yl)-5-(3-carboxymethoxyphenyl)-2-(4-sulfophenyl)-2H-tetrazolium
qRT-PCR: quantitative real-time polymerase chain reaction
GAPDH: glyceraldehydes-3-phosphate dehydrogenase
