# Supplementary material for: INC280 inhibits Wnt/β-catenin and EMT signaling pathways and its induce apoptosis in diffuse gastric cancer positive for c-MET amplification
Source: BMC Res Notes. 2019 Mar 11;12:125. doi: 10.1186/s13104-019-4163-x (PMC6419497; doi:10.1186/s13104-019-4163-x)
Supplement: Supplementary file 1 — Additional file 1: Table S1. Association of MET and RUNX3 expression with clinicopathological characteristics in 34 gastric cancer patients. Table S2. List of oligonucleotides for real-time PCR. Table S3. List of antibodies and its characteristic. [file 13104_2019_4163_MOESM1_ESM.doc]

**Table S1. Association of MET and RUNX3 expression with clinicopathological characteristics in 34 gastric cancer patients**

| Clinicopathological characteristics | MET expression | | *P*-value | RUNX3 expression | | *P*-value |
| --- | --- | --- | --- | --- | --- | --- |
|  | Fold change <2.0 (%) | Fold change >2.0 (%) |  | Fold change <2.0 (%) | Fold change >2.0 (%) |  |
| **Age** (median, range)  68.6 (44-87) years  >60 years old  <60 years old | 19(55.9)  8(23.5) | 6(17.7)  1(2.9) | 0.0002  0.027 | 24(70.6)  9(26.5) | 1(2.9)  0(0.0) | <0.001  <0.001 |
| **Sex**  Male  Female | 13(38.2)  14(41.2) | 6(17.7)  1(2.9) | 0.104  **<0.001** | 18 (52.9)  14(41.2) | 1(2.9)  1(2.9) | <0.001  <0.001 |
| **Differentiation**  Well differentiated  Moderate  Poor  Signet ring cell carcinoma  Mucinous  Mix | 0(0.0)  9(26.5)  12(35.3)  2(5.9)  3(8.8)  1(2.9) | 0(0.0)  0(0.0)  6(17.7)  0(0.0)  0(0.0)  1(2.9) | 1.000  0.002  0.168  0.493  0.239  1.000 | 0(0.0)  9(26.5)  18(52.9)  2(5.9)  2(5.9)  2(5.9) | 0(0.0)  0(0.0)  0(0.0)  0(0.0)  1(2.9)  0(0.0) | 1.000  0.002  **<0.001**  0.493  0.614  0.493 |
| **Ki-67**  High  Low  Int | 20(58.8)  5(14.7)  2(5.9) | 7(20.6)  0(0.0)  0(0.0) | 0.003  0.053  0.493 | 27(79.4)  4(11.8)  2(5.9) | 0(0.0)  1(2.9)  0(0.0) | **<0.001**  0.356  0.493 |
| **Lauren’s classification**  Diffuse  Intestinal  Mixed | 12(35.3)  6(17.7)  9(26.5) | 3(8.8)  1(2.9)  3(8.8) | 0.017  0.105  0.109 | 14(41.2)  7 (20.6)  12(35.3) | 1(2.9)  0(0.0)  0(0.0) | **<0.001**  0.011  **<0.001** |
| **TNM class**  I**A**  I**B**  IIA  IIB  IIIA  IIIB  IIIC  IV | 0(0.0)  1(2.9)  4(11.8)  4(11.8)  3(8.8)  9(26.5)  6(17.7)  0 (0.0) | 0(0.0)  0(0.0)  1(2.9)  0(0.0)  2(5.9)  1(2.9)  1(2.9)  2(5.9) | 1.000  1.000  0.356  0.114  1.000  0.013  0.105  0.493 | 0(0.0)  1(2.9)  5 (14.7)  3(8.8)  5(14.7)  10(29.4)  7(20.6)  2(5.9) | 0(0.0)  0(0.0)  0(0.0)  1(2.9)  0(0.0)  0(0.0)  0(0.0)  0(0.0) | 1.000  1.000  0.053  0.614  0.053  **<0.001**  **0.011**  0.493 |
| **Recurrence** | 8/9(88.9) | 1/9(11.1) | 0.003 | **9/9 (100)** | **0/9 (0.0)** | **<0.001** |

**Table S2. List of oligonucleotides for real-time PCR**

| Gene | Sequence (5’🡪3’) |
| --- | --- |
| RUNX3 | F: TTT CAC CCT GAC CAT CAC TG  R: GTC TGG TCC TCC AGC TTC TG |
| c-MET | F: AAG AGG GCA TTT TGG TTG TG  R: GAT GAT TCC CTC GGT CAG AA |
| ß-catenin | F: TCA TGC GTT CTC CTC AGA TG  R: CTC ACG ATG ATG GGA AAG GT |
| WNT1 | F: TCC TCC ACG AAC CTG CTT AC  R: CGG ATT TTG GCG TAT CAG AC |
| GSK3ß | F: GAA CTC CAA CAA GGG AGC AA  R: GGG TCG GAA GAC CTT AGT CC |
| SNAIL | F: CCT CCC TGT CAG ATG AGG AC  R GCC TCC AAG GAA GAG ACT GA |
| c-MYC | F: TCA AGA GGC GAA CAC ACA AC  R: GGC CTT TTC ATT GTT TTC CA |
| CCND1 | F: GAT CAA GTG TGA CCC GGA CT  R: TCC TCC TCT TCC TCC TCC TC |
| CD31 | F: GTG CTG CAA TGT GCT GTG AA  R: GCT TGG TCC AAA ATG CCT GG |
| CD44s | F: AAA GGA GCA GCA CTT CAG GA  R: TGT GTC TTG GTC TCT GGT AGC |
| ECAD | F: TGG GCC AGG AAA TCA CAT CC  R: GGC ACC AGT GTC CGG ATT AA |
| Human/Mouse GAPDH | F: TTC ACC ACC ATG GAG AAG GC  R: GGC ATG GAC TGT GGT CAT GA |

**Table S3. List of antibodies and their characteristic**

| Antigen | Antibody | Experimental conditions |
| --- | --- | --- |
| phospho-c-MET (Tyr1234/1235) | Cell Signaling Technology (#3077) | WB (1:1000) |
| c-MET | Cell Signaling Technology (#4560) | WB (1:1000) |
| phospho-Akt (Ser473) | Cell Signaling Technology (#4060) | WB (1:1000) |
| Akt | Epitomics (#1085-1) | WB (1:1000) |
| phospho-Erk1/2 (Thr202/Tyr204) | Cell Signaling Technology (#9101) | WB (1:1000) |
| Erk1/2 | Cell Signaling Technology (#4696) | WB (1:1000) |
| phospho-ß-catenin (Ser33/37/Thr41) | Cell Signaling Technology (#4270) | WB (1:1000) |
| ß-catenin | BD Biosciences (#610153) | WB (1:1000)  IF (1:100) |
| GSK-3ß | Santa Cruz Biotechnology (sc-7291) | WB (1:1000) |
| RUNX3 | Santa Cruz Biotechnology (sc-101553) | WB (1:1000) |
| Snail | Cell Signaling Technology (#3879) | WB (1:1000) |
| c-MYC | Santa Cruz Biotechnology (sc-40) | WB (1:1000) |
| ß-actin | Santa Cruz Biotechnology (sc47778) | WB (1:1000) |

WB, Western blot
