# Supplementary figures and images for: INC280 inhibits Wnt/β-catenin and EMT signaling pathways and its induce apoptosis in diffuse gastric cancer positive for c-MET amplification
Source: BMC Res Notes. 2019 Mar 11;12:125. doi: 10.1186/s13104-019-4163-x (PMC6419497; doi:10.1186/s13104-019-4163-x)

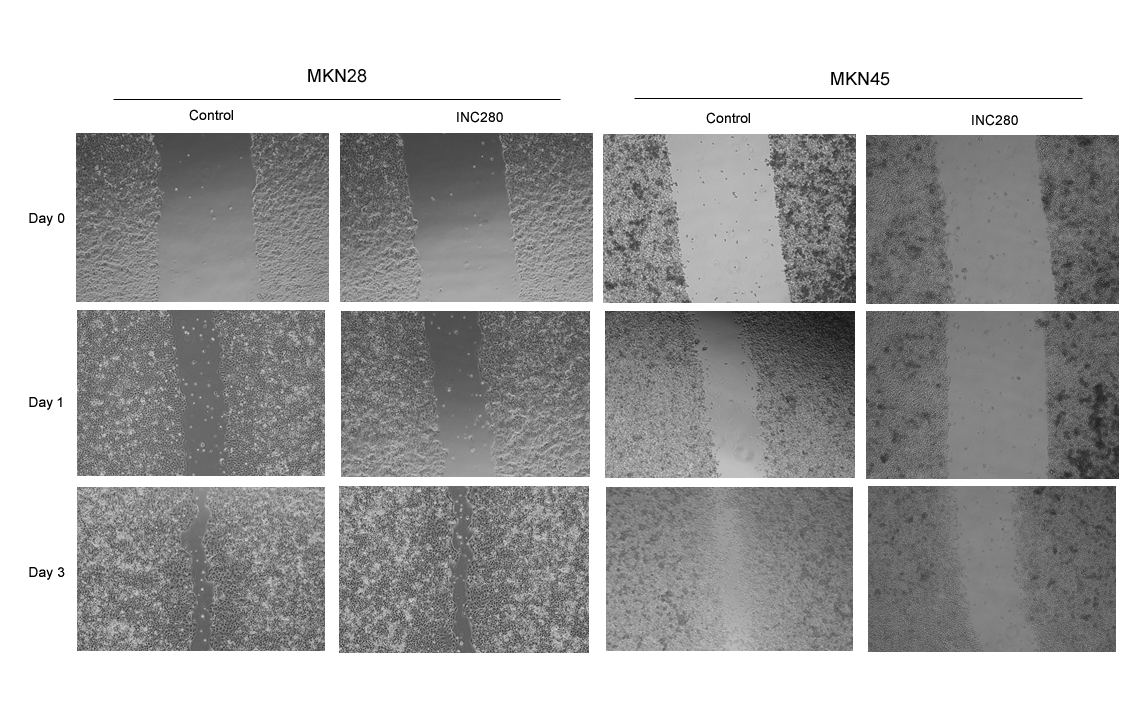


**Figure S1.**

**A**


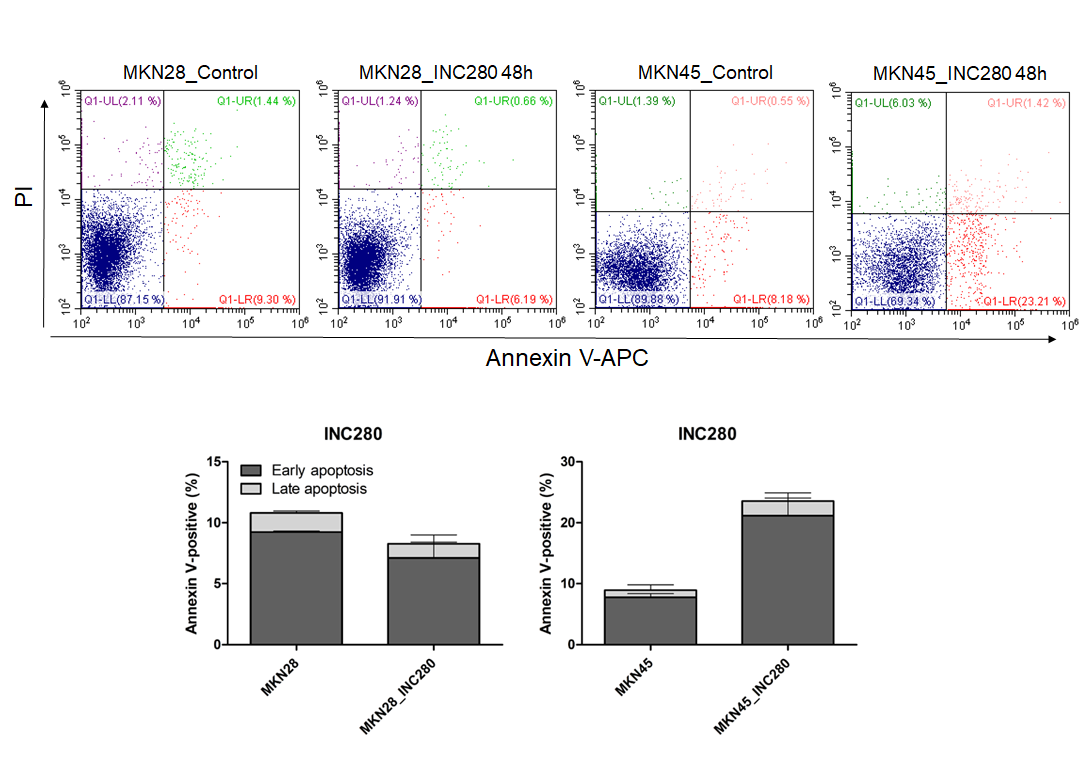


**B**

**
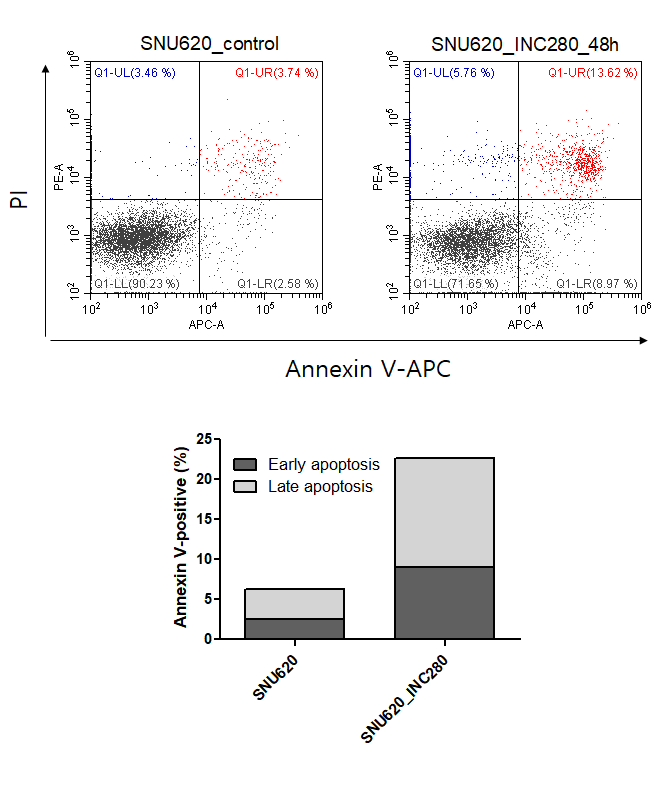
**

**Figure S2.**

Supplement: Supplementary file 2 — Additional file 2: Figure S1. Wound-healing assay was used to assess the effect of INC280 on the migration ability of MKN28 and MKN45 cells. INC280-treated MKN45 cells showed suppressed migration ability compared with INC280-treated MKN28 cell lines. Figure S2. Effect of INC280 on cell death in MKN28, SNU620, and MKN45 cells. Flow cytometric analysis of INC280-induced apoptosis in A: MKN28 and MKN45, B: SNU620 cells. PI, propidium iodide. [file 13104_2019_4163_MOESM2_ESM.doc]
